# The inhibition effect mechanisms of four scale inhibitors on the formation and crystal growth of CaCO_3_ in solution

**DOI:** 10.1038/s41598-019-50012-7

**Published:** 2019-09-16

**Authors:** Changjun Li, Chaoyi Zhang, Wuping Zhang

**Affiliations:** 1 School of Petroleum and Natural Gas Engineering, Southwest Petroleum University, Chengdu, 610500 China; 2 CNPC Key Laboratory of Oil & Gas Storage and Transportation, Southwest Petroleum University, Chengdu, 610500 China; 3 Institute of Chemical Engineering, East China University of Science and Technology, Shanghai, 200237 China

**Keywords:** Molecular dynamics, Computational chemistry

## Abstract

The experimentation, molecular dynamics simulation and DFT calculation were used to study the inhibition effects of four scale inhibitors, including polyacrylic acid (PAA), hydrolyzed polymaleic anhydride (HPMA), polyepoxysuccinic acid (PESA) and polyaspartic acid (PASP), on formation and crystal growth of CaCO_3_ in solutions. According to concentrations of Ca^2+^ in solutions, the sequence of inhibition effects of scale inhibitors on formation of CaCO_3_ in the solution was PESA > PASP > HPMA > PAA. Characterization of CaCO_3_ crystals by XRD and a laser particle size analyzer indicated that the sequence of inhibition effects of scale inhibitors on crystal growth of CaCO_3_ in solutions was PESA > HPMA > PASP > PAA. Interaction energies between the scale inhibitor molecule and Ca^2+^, and between the scale inhibitor molecule and the CaCO_3_ (104) surface indicated that the difference of the inhibition effects was derived from the difference in the interaction energy. The results of DFT calculation indicated that the difference between the interaction energies of these inhibitors and Ca^2+^ was derived from differences of number and the Mulliken population values of the chemical bonds which formed between the inhibitor molecule and Ca^2+^ and between the inhibitor molecule and the CaCO_3_ surface.

## Introduction

Produced water in gas fields is one of the by-products of natural gas production. Since the produced water contains a variety of ions, especially calcium, the formation of water-insoluble compounds is common via various chemical reactions. The most common compound is CaCO_3_, forming undesirable scale. Since the produced water is usually separated from the gas in the separator and then passed through the sewage pipe into other equipment, the sewage pipe is the most severely scaled area. The main target of scale inhibition in gas fields is also the sewage in sewage pipes.

For four common non-phosphorus scale inhibitors, including polyacrylic acid (PAA), hydrolyzed polymaleic anhydride (HPMA), polyepoxysuccinic acid (PESA) and polyaspartic acid (PASP), the scale inhibition effects on CaCO_3_ in solution mainly include two aspects. One aspect is to inhibit the formation of CaCO_3_. When the scale inhibitor is present in the solution, the Ca^2+^ concentration increases with the increase of the concentration of the scale inhibitor. Therefore, the amount of formed CaCO_3_ decreases^1–6^. The other aspect is to inhibit the growth of CaCO_3_ crystals. Calcite is the most stable crystal structure of CaCO_3_ in general, and the most common solid in scale. PAA, HPMA, PESA and PASP can all be adsorbed to the main growth surface of the calcite crystal, therefore inhibiting the growth of crystals and resulting in the destruction of the regular shape of the calcite crystal^2–10^. This leads to the weakening of the crystal stability. The scale inhibition effect mechanisms among these scale inhibitors were also compared. HPMA has higher scale inhibition effect than PAA^3^, and PESA has better scale inhibition effect than PAA, HPMA and PASP^11,12^.

The previously reported research focused on describing the inhibitory effects from experimental results and lacked research on the mechanisms of inhibition. In this paper, we first evaluated the scale inhibition effect of four scale inhibitors based on the experimental results. We then established a molecular dynamics simulation model to calculate the interaction energies between the scale inhibitor molecules and Ca^2+^, as well as the scale inhibitor molecules and the calcite surface, which can illustrate the reason for the scale effects of scale inhibition. Finally, we implemented a DFT calculation for the model, included the number of bonds, and the Mulliken Population value of the bonds to explain the reason for the difference in adsorption energy.

## Experiment Methods

### Materials

The research inspiration in this paper is a gas field sewage station in Shandong, China. The main scaled ions in the sewage are Ca^2+^ and HCO_3_^−^, and the main component of the scale is CaCO_3_. Therefore, CaCl_2_ and NaHCO_3_ are used to form CaCO_3_. The experiment used CaCl_2_ and NaHCO_3_ that were analytically pure with the content of >96%, purchased from Sichuan Kelong Company. The concentrations of Ca^2+^ and HCO_3_^−^ in the sewage were 0.336 g/L and 0.696 g/L, respectively. The concentrations of CaCl_2_ and NaHCO_3_ in the solution were 0.933 g/L and 0.959 g/L after conversion, respectively.

The mass concentrations of four scale inhibitors including PAA, HPMA, PESA and PASP were all 50%. They were purchased from Kairui Company in Shandong, China. Each scale inhibitor was pre-diluted to 1 g/L with ultra pure water. In each group of the experiment, 10 mL inhibitor solution was poured into the ultra pure water (i.e., the concentration of the scale inhibitor in the test solution was 10 mg/L).

### Experimental procedures

The scale inhibition effects of the scale inhibitor on CaCO_3_ include the inhibition of formation and crystal growth of CaCO_3_. Therefore, the experiment was divided into two groups. The experimental temperature was set at 51 °C and the pH was 6.6–6.8. (The temperature and pH were the same operational conditions as the as at the sewage station).

#### Experiment 1: inhibiting the formation of CaCO_3_

To begin, 1 L UP water (without scale inhibitor) and 0.99 L UP water with added scale inhibitor were added to each beaker. Additionally, 30 mL mixed solution (contains HCl, ammonia-ammonium chloride buffer solution and UP water) was added to each beaker during the experiment to control the pH of solution and compensate for the evaporation loss. The beakers were placed on a magnetic stirrer and heat to 51 °C. 0.959 g NaHCO_3_ and 10 mL scale inhibitor were added to the solution. After stirring for 30 min, 0.933 g CaCl_2_ was added into solution and start the experiment. The experiment duration was 24 h. After the experiment was completed, wait time of 6 h was needed to allow the CaCO_3_ solid precipitate. The supernatant was poured into a Buchner funnel with double-layer filter paper for filtration. Each set of clear liquids was repeatedly filtered 3 times. The clear liquid after the third filtration was stored for examination. The experiment of inhibiting the formation of CaCO_3_ was repeat three times.

#### Experiment 2: inhibiting crystal growth of CaCO_3_

To begin, 1 L UP water (without scale inhibitor) and 0.99 L UP water with added scale inhibitor were added to each beaker. Additionally, 30 mL mixed solution (contains HCl, ammonia-ammonium chloride buffer solution and UP water) was added to each beaker during the experiment to control the pH of solution and compensate for the evaporation loss. The beakers were placed on a magnetic stirrer and brought to 51 °C. NaHCO_3_ and CaCl_2_ were added to the solution. After stirring for 30 min, the scale inhibitor was added to start the experiment. The experiment was left to react for 24 h. After the experiment completed, the turbid liquid was poured into a Buchner funnel with single-layer filter paper for filtration. After filtration, the filter paper containing the slurry of CaCO_3_ was placed in an oven (105 °C) for 6 h. Then the dry CaCO_3_ powder was stored for examination. The experiment of inhibiting crystal growth of CaCO_3_ was repeat three times.

## Molecular Models and Simulation Details

### Software and force field

In this study, the amorphous cell, Discover, Forcite, and Castep modules in Materials Studio 7.0 software were used. The amorphous cell module was used to create a mixed layer of water molecules and scale inhibitor molecules. The Discover module was used to minimize energy, while the Forcite module was used to run molecular dynamics simulation programs using the COMPASS force field^13–15^. The Castep module was used to calculate the bond number and the Mulliken population value between the scale inhibitor molecule and the surface. The functional used for these calculations is the GGA of Perdew, Burke, and Enzerhof (PBE)^16,17^.

### Molecular models

**Figure 1:**
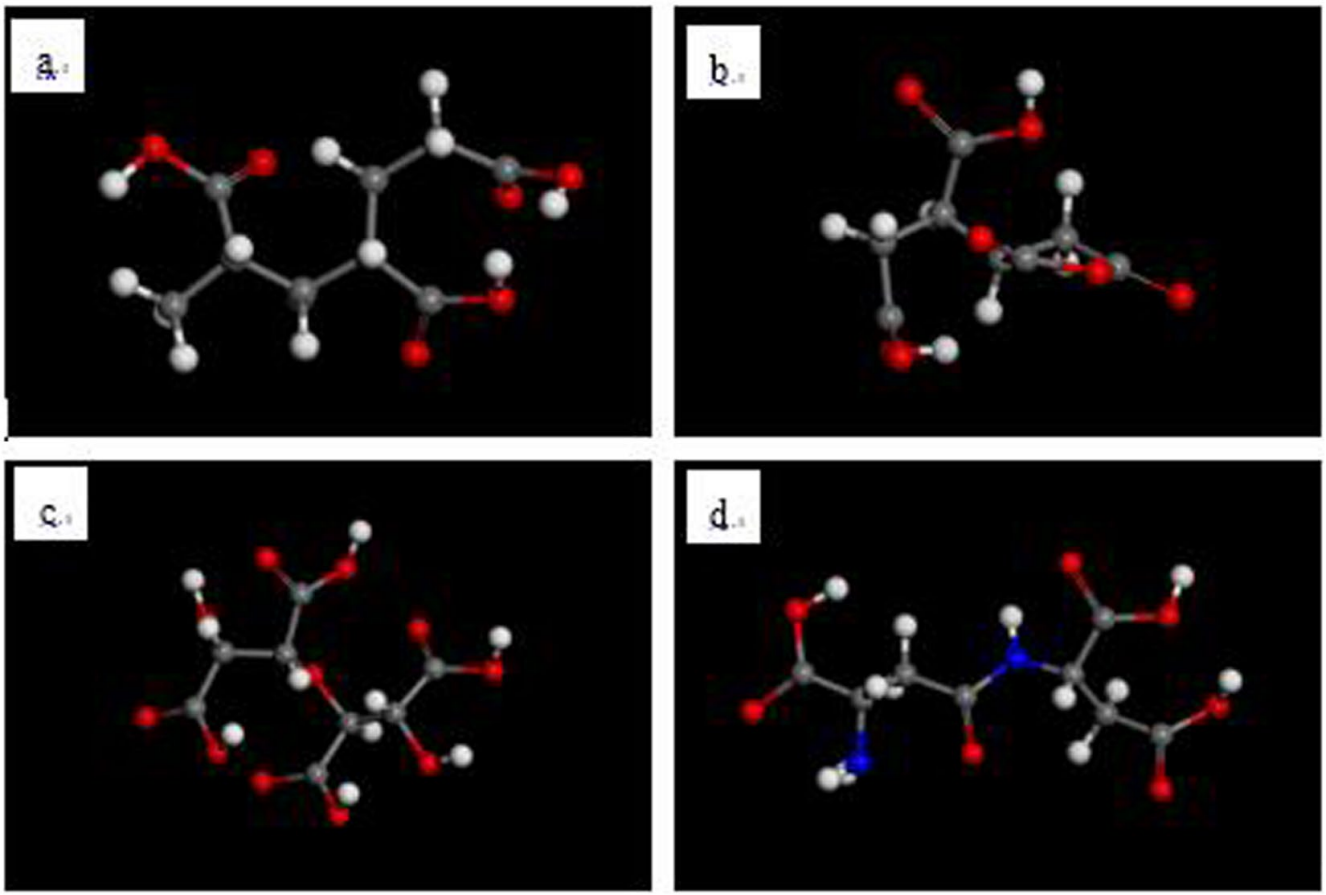
PAA (**a**), HPMA (**b**), PESA **(c**) and PASP (**d**) scale inhibitor models (red - O atom; white - H atom; gray - C atom; dark blue -N atom).

The four scale inhibitor molecules are drawn manually, as shown in Fig. 1.

#### Model for inhibiting formation of CaCO_3_

**Figure 2:**
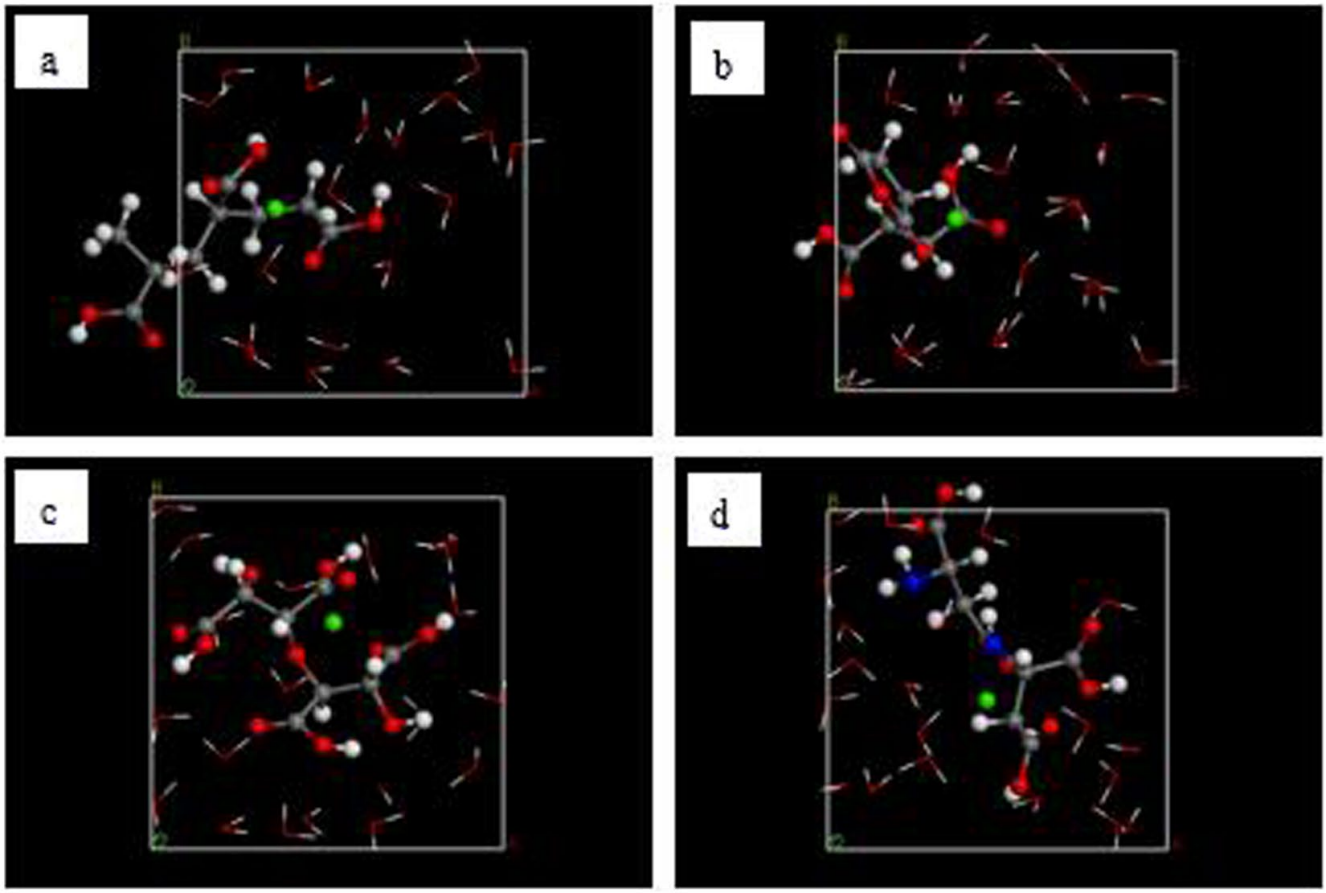
Initial models of interaction between Ca^2+^ and (**a**) PAA, (**b**) HPMA, (**c**) PESA, and (**d**) PASP. (Red - O atom; white - H atom; gray - C atom; dark blue - N atom; green: Ca atom).

Since the scale inhibitors prevent the formation of CaCO_3_ based on the Ca^2+^ concentration, the interaction between the inhibitor molecules and Ca^2+^ can be used to evaluate the scale inhibition effect^2,18–20^. The model used to examine the inhibition of the formation of CaCO_3_ contained 1 scale inhibitor molecule, 1 Ca^2+^, and 20 water molecules and was built using the amorphous cell module in Materials Studio. The initial configuration of this model is shown in Fig. 2. In order to ensure the ionization of Ca in this model, its charge and force field were the same as those used for the Ca^2+^ in the CaCO_3_ molecule.

#### Model for inhibiting crystal growth of CaCO_3_

**Figure 3:**
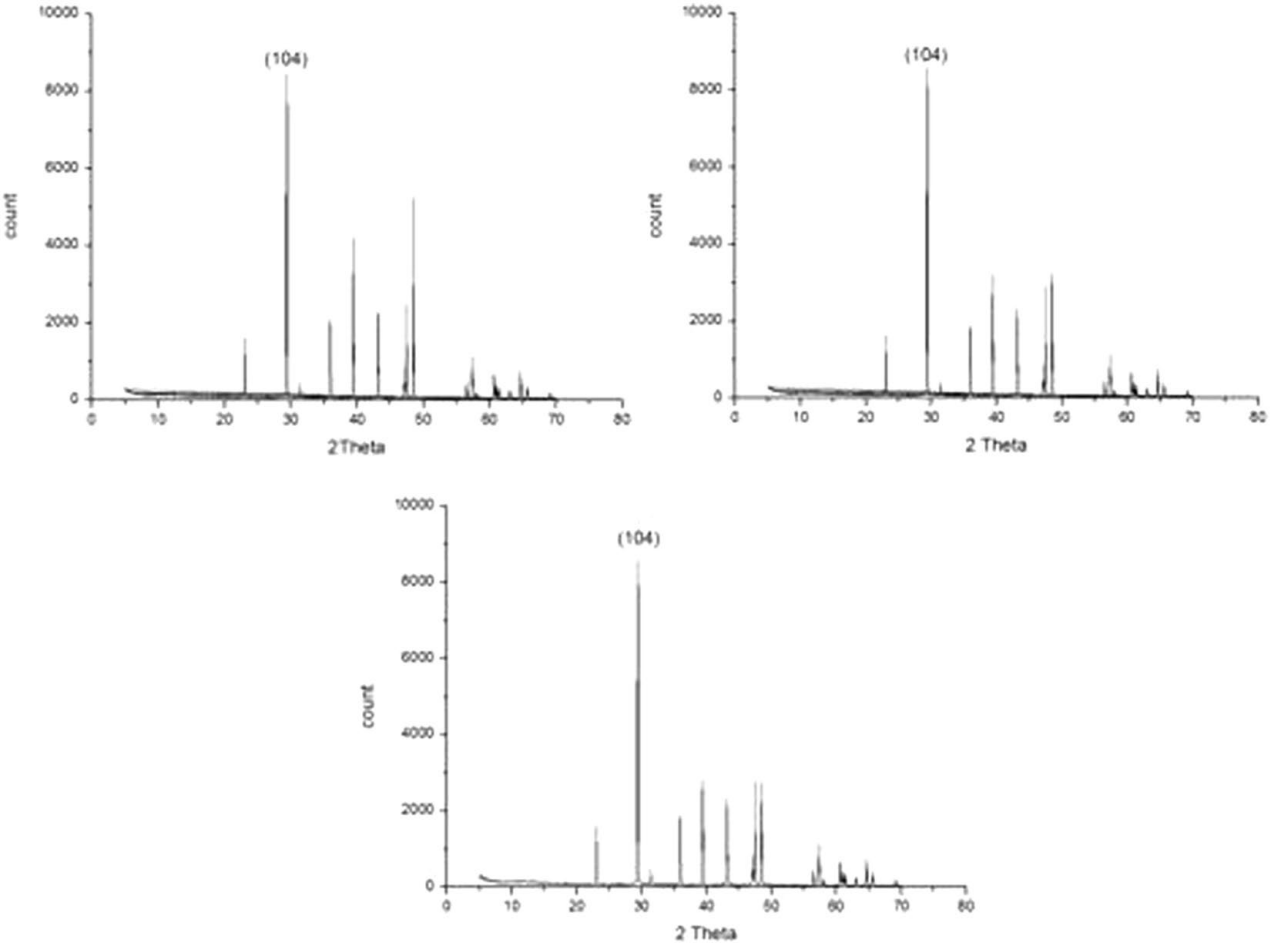
XRD of CaCO_3_ crystal formed in absence of scale inhibitors in three groups of experiment.

**Figure 4:**
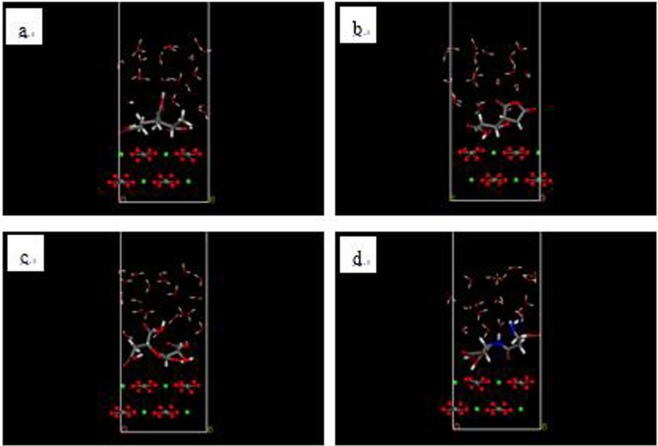
Initial models of interaction between CaCO_3_ (104) surface and (**a**) PAA, (**b**) HPMA, (**c**) PESA and (**d**) PASP (red - O atom; white - H atom; gray - C atom; dark blue - N atom; green: Ca atom).

As shown in Fig. 3, the X-ray diffraction (XRD) spectrum of CaCO3 shows that the peak corresponding to the (104) surface was significantly higher than that of the other surfaces, in the absence of scale inhibitors. Hence, the (104) surface is used as the surface of the CaCO_3_ crystal. The initial molecular models of the CaCO_3_ crystals were imported from a software database. The designated surface was cut to obtain the required adsorption surface. The a, b and c values of the (104) surface model of the established CaCO_3_ crystal were 8.09 Å, 9.98 Å and 37.91 Å, respectively. Since the rotation of CO_3_^2−^ had a large influence on the adsorption process, the Ca and C atoms in the crystal surface were set to the fixed state, and the O atom was set to the free state^21^. A mixed layer, using one scale inhibitor molecule and 20 water molecules, was created in the amorphous cell module and the a and b values were chosen to be identical to the surface model values. The surface model was combined with the mixed layer by using thebuild layers program in Materials Studio software. The Finitial model for inhibiting the CaCO_3_ crystal growth is shown in Fig. 4.

### Simulation conditions

**Figure 5:**
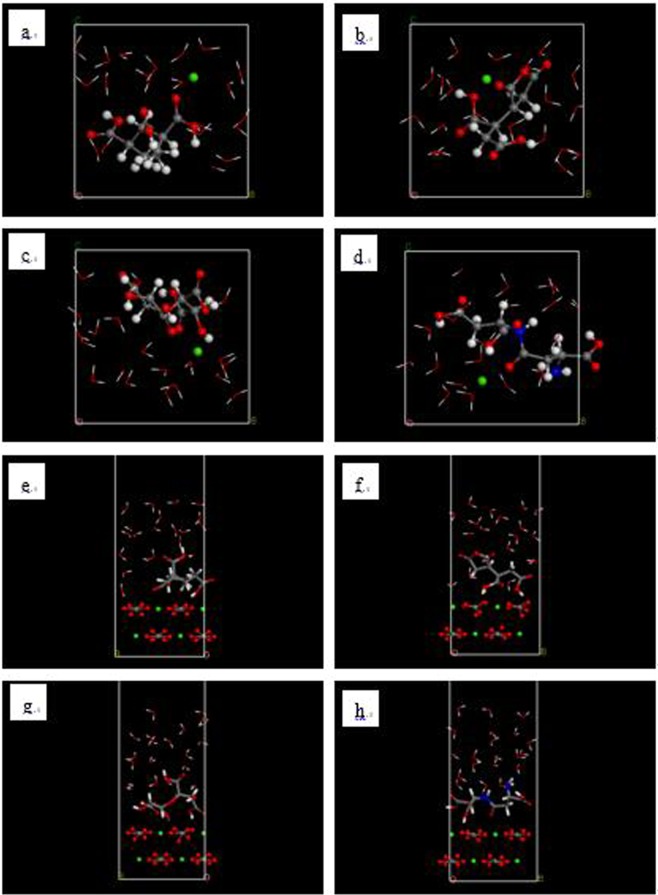
Final models of interaction between Ca^2+^ (**a–d**) and between CaCO_3_ (104) surface (**e–h**) and (**a**) PAA, (**b**) HPMA, (**c**) PESA and (**d**) PASP (red - O atom; white - H atom; gray - C atom; dark blue - N atom; green: Ca atom).

Once the models were created, the energy of each was minimized using the smart minimizer, which includes steepest descent, conjugate gradient and Newton methods. The convergence of all methods was set at 10^−7^. The Forcite module was used to perform molecular dynamics simulations. The temperature was set to 324 K (i.e., 51 °C), and 20 million steps in the NVT ensemble were performed using a Berendsen thermostat. After the molecular dynamics simulations completed, the final state of the model is shown in Fig. 5. Finally, the Castep module of Materials Studio was used to perform DFT calculations. In this module, the PBE functional was chosen, and Fine was selected as Quality.

## Results and Discussion

### Scale inhibitor and Ca^2+^

The clear liquid was placed in an ion chromatograph (ICS-5000, Thermofisher Scientific CO., USA) for detection, and the obtained Ca^2+^ concentration is shown in Table 1.

**Table 1:** Concentration of Ca^2+^ (mg/L) in different solutions.

Solution		No inhibitor	PAA	HPMA	PESA	PASP
First group of experiment	Concentration of Ca^2+^	110.2537	286.1465	298.1426	321.1026	305.1236
	SD	0.0003	0.0007	0.0015	0.0010	0.0022
	RSD(%)	0.6331	0.7606	0.9535	0.4726	0.8004
Second group of experiment	Concentration of Ca^2+^	107.1461	287.0162	295.2617	319.1497	309.2516
	SD	0.0019	0.0003	0.0025	0.0002	0.0011
	RSD(%)	0.7432	0.1586	0.7201	0.1053	0.4852
Third group of experiment	Concentration of Ca^2+^	106.9107	290.8844	295.2008	319.9769	310.0763
	SD	0.0014	0.0016	0.0007	0.0021	0.0015
	RSD(%)	0.5219	0.9195	0.2653	0.6817	0.4551
Average concentration of Ca^2+^		108.1035	288.0157	296.2017	320.0764	308.1505

As shown in Table 1, the concentration of Ca^2+^ in the solution containing no scale inhibitor was significantly lower than that in the solution containing scale inhibitors, indicating that most of the Ca^2+^ was formed as a precipitate of CaCO_3_ in absence of scale inhibitors. Most Ca^2+^ remained in a free state in presence of scale inhibitors. The sequence of Ca^2+^ concentration in solutions containing different scale inhibitors was PESA > PASP > HPMA > PAA. Therefore, the sequence of effects of inhibiting the formation of CaCO_3_ was PESA > PASP > HPMA > PAA.

### Scale inhibitor inhibiting crystal growth of CaCO_3_

**Figure 6:**
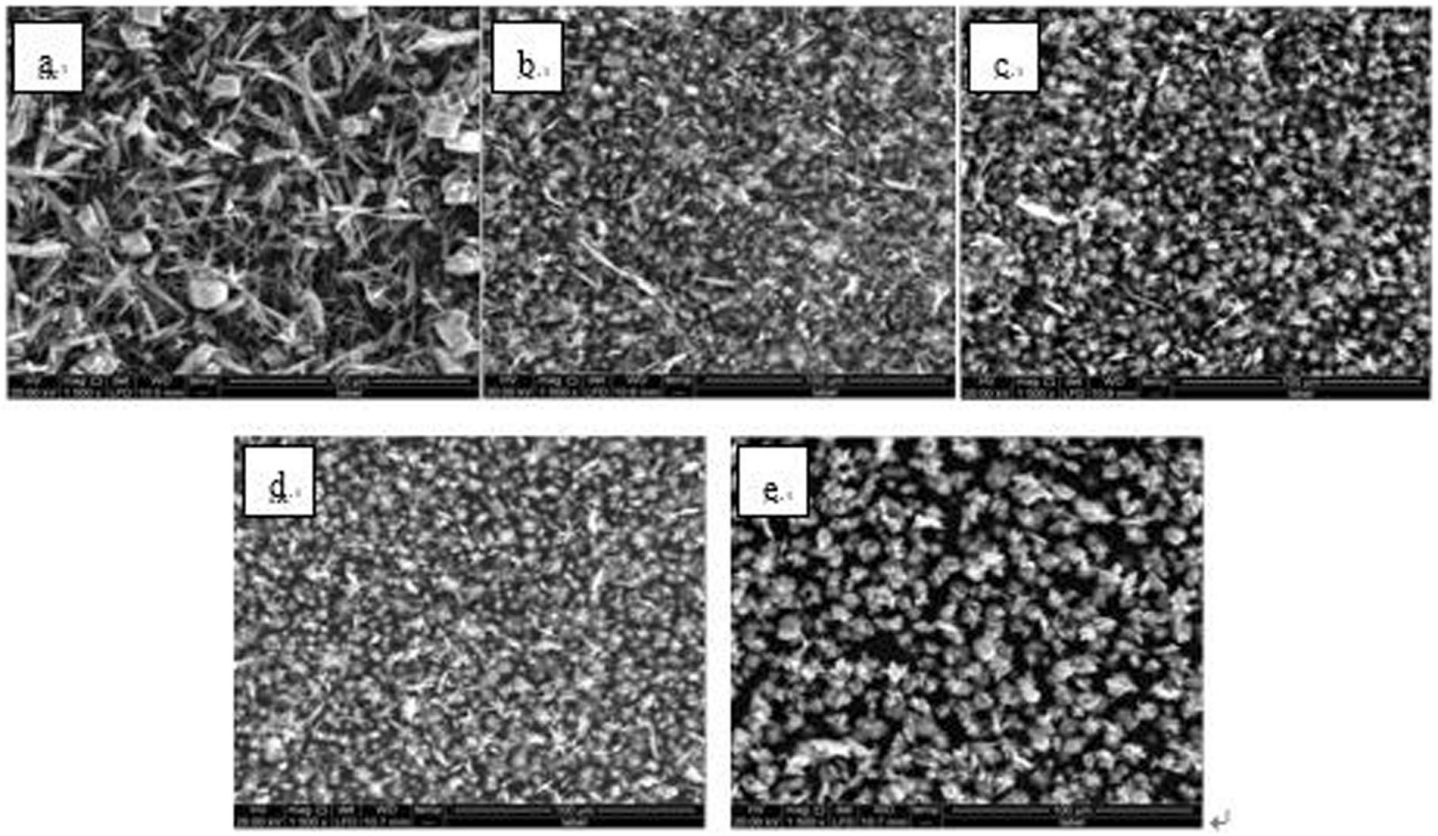
Morphologies of CaCO_3_ crystals with various added scale inhibitor solutions in first group of experiment. (**a**) No scale inhibitor; (**b**) containing PAA; (**c**) containing HPMA; (**d**) containing PESA; (**e**) containing PASP.

**Figure 7:**
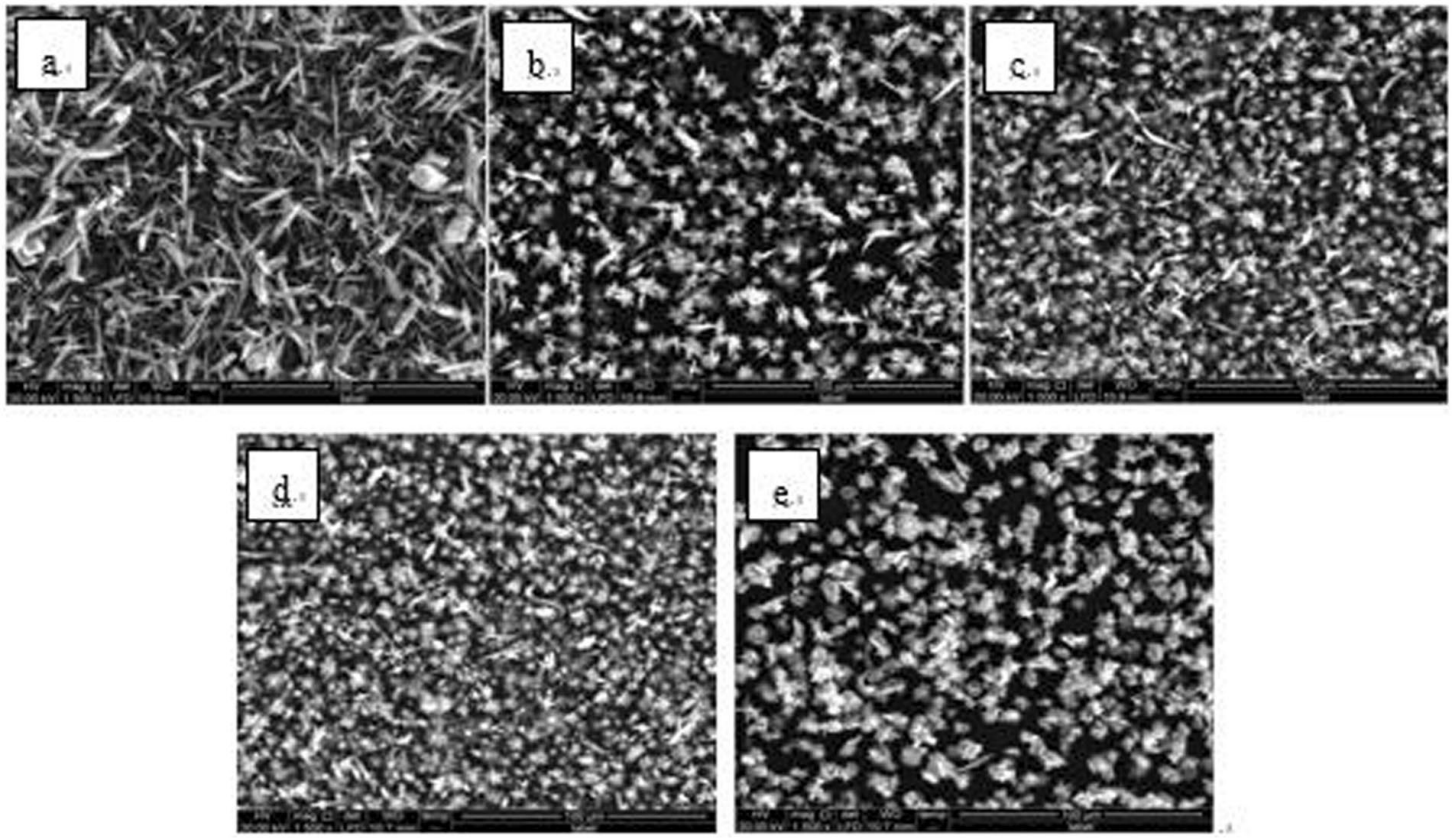
Morphologies of CaCO_3_ crystals with various added scale inhibitor solutions in second group of experiment. (**a**) No scale inhibitor; (**b**) containing PAA; (**c**) containing HPMA; (**d**) containing PESA; (**e**) containing PASP.

**Figure 8:**
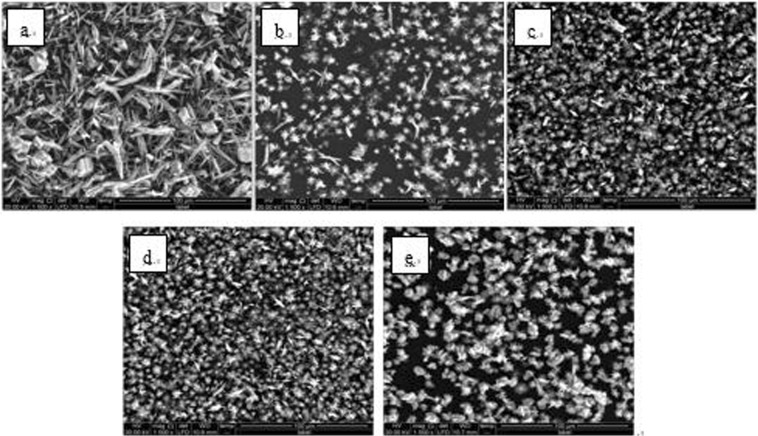
Morphologies of CaCO_3_ crystals with various added scale inhibitor solutions in third group of experiment. (**a**) No scale inhibitor; (**b**) containing PAA; (**c**) containing HPMA; (**d**) containing PESA; (**e**) containing PASP.

**Figure 9:**
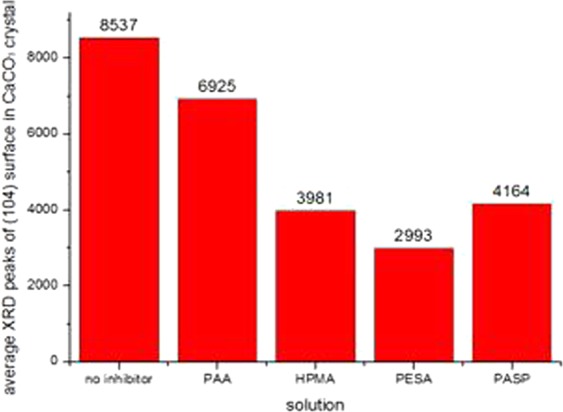
Comparison of average XRD peaks of (104) surface in CaCO_3_ crystal.

**Figure 10:**
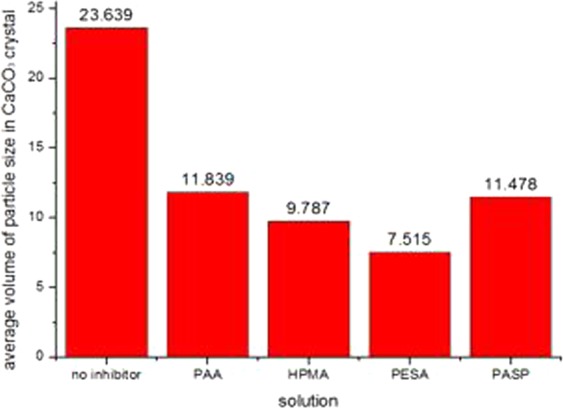
Comparison of average volume of particle sizes in CaCO_3_ crystals.

The experimentally obtained powders were examined by SEM (Quanta 250, FEI Co., USA), XRD (D8 ADVANCE, Bruker AXS CO., Germany) and laser particle analyzer (HYDRO2000 (APA2000), Malvern CO. UK), respectively. The CaCO_3_ crystal morphology are shown in Figs 6–8. The XRD peak of the CaCO_3_ (104) surface and the average volume of particle size of CaCO_3_ crystal are shown in Figs 9 and 10 and Table 2, respectively.

**Table 2:** The XRD peaks of (104) surface and volume of particle sizes of CaCO_3_ crystals.

Solution		No inhibitor	PAA	HPMA	PESA	PASP
First group of experiment	XRD peaks of (104) surface	8451	6865	4088	3030	4128
	volume of particle sizes	23.536	11.999	9.735	7.633	11.279
Second group of experiment	XRD peaks of (104) surface	8591	7037	3959	3007	4358
	volume of particle sizes	23.621	11.741	9.786	7.611	11.496
Third group of experiment	XRD peaks of (104) surface	8569	6873	3896	2942	4006
	volume of particle sizes	23.761	11.778	9.841	7.302	11.659
Average	XRD peaks of (104) surface	8537	6925	3981	2993	4164
	volume of particle sizes	23.639	11.839	9.787	7.515	11.478

As shown in Figs 6–8, in absence of scale inhibitors, the CaCO_3_ crystal mainly exhibited long needle-like and hexahedral shapes. In presence of scale inhibitors, the particle size of the CaCO_3_ crystal reduced significantly, and the CaCO_3_ crystal exhibited short needle-like and irregular polyhedron shapes. As shown in Figs 9 and 10 and Table 2, the XRD peak of the (104) surface and the CaCO_3_ crystal average volume particle size in presence of scale inhibitors were significantly smaller than those in absence of scale inhibitors.

Also as shown in Figs 6–10 and Table 2, in presence of scale inhibitors, the crystal growth of the (104) surface of CaCO_3_ was suppressed, the overall growth rate of the crystal was decelerated, and the average volume particle size was reduced. The sequences of both XRD peak and the volume average particle size in solutions containing different scale inhibitors were PESA < HPMA < PASP < PAA. Hence, the sequence of effects of inhibiting the CaCO_3_ growth was PESA > HPMA > PASP > PAA.

### Interaction energy calculations

According to the experimental results, the interaction between the scale inhibitor molecule and Ca^2+^ and the adsorption of the scale inhibitor molecule on the CaCO_3_ (104) surface were the main factors inhibiting formation and crystal growth of CaCO_3_, respectively. The equations used to calculate the interaction energy between the scale inhibitor molecules and the Ca^2+^, as well as the CaCO_3_(104) surface are expressed as^22,23^:

1 $$\Delta {E}_{1}={E}_{{\rm{Ca}}+{\rm{inhi}}}-({E}_{{\rm{Ca}}}+{E}_{{\rm{inhi}}})$$

2 $$\Delta {E}_{2}={E}_{{\rm{surf}}+{\rm{inhi}}}-({E}_{{\rm{surf}}}+{E}_{{\rm{inhi}}})$$

where Δ*E*_1_ refers to the interaction energy between Ca^2+^ and the scale inhibitor molecule, Δ*E*_2_ refers to the interaction energy between the CaCO_3_ (104) surface and the scale inhibitor molecule, *E*_ca+inhi_ refers to the energy in the model in presence of both Ca^2+^ and scale inhibitor molecules, *E*_surf+inhi_ refers to the energy in the model in presence of both the CaCO_3_ (104) surface and scale inhibitor molecules, *E*_ca_, *E*_inhi_, and *E*_surf_ refer to the energy in the model in presence of Ca^2+^, scale inhibitors and the CaCO_3_ (104) surface, respectively. The interaction energies between Ca^2+^ and the CaCO_3_ (104) surface with the scale inhibitor molecules are shown in Tables 2 and 3, respectively.

**Table 3:** Interaction energies between the four inhibitor molecules and Ca^2+^. All values are in kcal/mol.

Inhibitor	*E*_ca+inhi_	*E*_ca_	*E*_inhi_	Δ*E*_1_
PAA	309.86	399.304	−48.985	−40.459
HPMA	268.096	400.567	−90.656	−41.815
PESA	294.322	372.776	17.001	−95.455
PASP	271.749	384.14	−48.586	−63.805

All of the Δ*E*_1_ and Δ*E*_2_ values in Tables 3 and 4 are negative, indicating that the interactions between the inhibitors and Ca^2+^ and CaCO_3_ (104) are spontaneous. Comparing the values of Δ*E*_1_ and Δ*E*_2_, the sequences of interaction energies between Ca^2+^ and scale inhibitor molecules and the CaCO_3_ (104) surface are PESA < PASP < HPMA < PAA and PESA < HPMA < PASP < PAA, respectively.

**Table 4:** Interaction energy between the four inhibitor molecules and the CaCO_3_ (104) surface. All values are in kcal/mol.

Inhibitor	*E*_surf+inhi_	*E*_surf_	*E*_inhi_	Δ*E*_2_
PAA	−5922.096	−5833.991	−26.24	−61.865
HPMA	−5975.804	−5817.605	−77.32	−80.879
PESA	−5909.894	−5830.47	7.734	−87.158
PASP	−5971.353	−5834.883	−61.461	−75.009

When the value of the interaction energy is more negative, the interaction modeled is more stable and the intensity of the action was higher. Therefore, the sequence of interaction strengths between Ca^2+^ and the scale inhibitor molecules is PESA > PASP > HPMA > PAA. The interaction between Ca^2+^ and scale inhibitor molecules was the main reason for Ca^2+^ to be in a free state. The inhibition of CaCO_3_ formation by each scale inhibitor seen experimentally correlates with the sequence of interaction strengths between Ca^2+^ and the scale inhibitor molecules.

The sequence of interaction strengths between the CaCO_3_ (104) surface and the scale inhibitor molecules is PESA > HPMA > PASP > PAA. If the bonding strength between the scale inhibitor and CaCO_3_ (104) surface was higher, the active growth point of the surface was occupied by the scale inhibitor molecule instead of the CaCO_3_ molecule, which resulted in the surface growth rate decreasing and further resulted in the growth rate of CaCO_3_ crystal decreasing. Therefore, the inhibition of CaCO_3_ crystal growth by each scale inhibitor seems to follow the sequence of interaction strengths between the CaCO_3_ (104) surface and the scale inhibitor molecules.

### DFT calculations

The interaction between the two components was proportional to the number of chemical bonds formed between the two interacting components and the Mulliken population value of the bonds. The interaction energy was inversely proportional to the number of chemical bonds and the Mulliken population value of the bonds. The chemical bonds formed, bond lengths, and Mulliken populations for the interaction between the scale inhibitor molecule and the Ca^2+^ and the CaCO_3_ (104) surface are shown in Tables 5 and 6, respectively.

**Table 5:** Chemical bonds, Mulliken population values of bonds, and bond lengths between Ca^2+^ and scale inhibitor molecules.

Inhibitor	Bond	Population	Length (Å)
PAA	Ca1-O2	0.02	3.13456
HPMA	Ca1-O6	0.11	2.42149
PESA	Ca1-O2	0.09	2.45118
	Ca1-O11	0.1	2.51438
	Ca1-O8	0.06	2.62985
	Ca1-O3	0.03	3.24524
PASP	Ca1-O8	0.12	2.41995

**Table 6:** Chemical bonds, Mulliken population values of bonds, and bond lengths formed between the CaCO_3_ (104) surface and scale inhibitor molecules.

Inhibitor	Bond	Population	Length (Å)
PAA	H10-O12	0.11	1.42513
	Ca5-O31	0.04	2.31432
HPMA	H8-O24	0.17	1.36346
	Ca8-O28	0.06	2.87023
	Ca5-O27	0.05	2.91259
	Ca5-O29	0.02	3.08848
PESA	H9-O10	0.11	1.50917
	H11-O12	0.08	1.60188
	Ca4-O29	0.08	2.3255
	Ca8-O34	0.06	2.33118
	Ca5-O35	0.08	2.4813
PASP	H13-O12	0.13	1.39291
	Ca8-O32	0.12	2.3861

As shown in Table 5, the bonds formed between the Ca^2+^ and the scale inhibitor molecules were formed by Ca^2+^ and O atoms in the scale inhibitor molecules^24^. PESA and Ca^2+^ formed four Ca-O bonds, the most bonds formed compared to the other three inhibitors. The interaction energy value between PESA and Ca^2+^ was the lowest of any inhibitor, therefore, the interaction was the strongest. The other three scale inhibitors only formed one Ca-O bond with Ca^2+^. The sequences of the Mulliken population values of the bonds and interaction intensities for the other three inhibitors are both PASP > HPMA > PAA. The sequence of interaction energy values is PASP < HPMA < PAA. According to the number of bonds formed and the Mulliken population values of the bond(s), the sequence of interaction strengths between the scale inhibitors and Ca^2+^ was PESA > PASP > HPMA > PAA, and the sequence of interaction energy values was PESA < PASP < HPMA < PAA.

As shown in Table 6, two types of chemical bonds are formed between the scale inhibitor molecules and the CaCO_3_ (104) surface. One bond formed is an O-H bond, formed by a H atom in the scale inhibitor and an O atom on the surface, while the other is a Ca-O bond, formed by an O atom in the scale inhibitor and a Ca atom on the surface of the crystal. PESA formed five bonds (two H-O bonds, three Ca-O bonds) with the surface, and HPMA formed four bonds (one H-O bond and three Ca-O bonds) with the surface. Since the number of O-H bonds and the Mulliken population values of Ca-O bonds formed by PESA and the surface were higher than those formed by HPMA and the surface, the interaction strength between PESA and the surface is higher than between HPMA and the surface. Both PASP and PAA formed only two bonds (1 H-O bond, 1 Ca-O bond) with the surface. Therefore, the interaction strengths between PASP and PAA with the surface are weaker than those between PESA and HPMA with the surface. By comparing the Mulliken population values of the same type of chemical bonds, it can be concluded that the strength between PASP and the surface is higher than that between PAA and the surface. According to the number of bonds formed and the Mulliken population values of the bonds, the sequence of interaction strengths between the scale inhibitors and the CaCO_3_ (104) surface is PESA > HPMA > PASP > PAA, while the sequence of interaction energy values is PESA < HPMA < PASP < PAA.

## Conclusions

In this study, the mechanism of inhibition effects of PAA, HPMA, PESA and PASP on the formation and crystal growth of CaCO_3_ in the solution were studied. According to the experimental results, the sequence of inhibition effects of scale inhibitor on formation of CaCO_3_ is PESA > PASP > HPMA > PAA, while the sequence of inhibitory effects on crystal growth of CaCO_3_ is PESA > HPMA > PASP > PAA. Calculating the interaction energies between the scale inhibitor molecules and Ca^2+^ as well as the CaCO_3_ (104) surface shows that the higher inhibition effect is derived from lower interaction energy values. DFT calculations indicate that lower interaction energy values are derived from the formation of a larger number of chemical bonds with higher Mulliken population values between the scale inhibitor and the Ca^2+^, as well as between scale inhibitor molecules and the CaCO_3_ (104) surface. According to the mechanism of the four common inhibitors, the inhibition effects of other inhibitors could be evaluated by similar means in the future.
